# The efficacy and safety of atherectomy combined with drug-coated balloon angioplasty vs. drug-coated balloon angioplasty for the treatment of lower extremity artery disease: a systematic review and meta-analysis

**DOI:** 10.3389/fcvm.2024.1472064

**Published:** 2024-09-27

**Authors:** Margareta Ginanti Ratna Indraswari Suriyanto, Raymond Pranata, William Kamarullah, Iwan Cahyo Santosa Putra, Dendi Puji Wahyudi, Giky Karwiky, Teddy Arnold Sihite, Mohammad Rizki Akbar, Januar Wibawa Martha, Syarief Hidayat

**Affiliations:** Department of Cardiology and Vascular Medicine, Faculty of Medicine, Universitas Padjadjaran, Hasan Sadikin General Hospital, Bandung, Indonesia

**Keywords:** directional atherectomy, drug-coated balloon angioplasty, lower extremity artery disease, atherectomy, angioplasty

## Abstract

**Background:**

This study aimed to systematically evaluate the efficacy and safety of atherectomy followed by drug-coated balloon angioplasty (A-DCB) in comparison with drug-coated balloon (DCB) angioplasty alone for the treatment of lower extremity artery disease (LEAD).

**Methods:**

Systematic literature search was performed using several online databases including MEDLINE (via PubMed), Europe PMC, and ScienceDirect databases from inception until February 21st, 2024. We included all studies comprised three main variables including A-DCB, DCB, and LEAD. The primary outcomes were primary patency and target lesion revascularization (TLR). Whereas secondary outcomes were all-cause mortality, post-procedural complications, and clinical characteristics.

**Results:**

A total of 15 studies (10 cohort studies and 5 randomized controlled trials studies) consisting of 1,385 participants with mean age 68.7 ± 8.9 were included. In comparison with DCB alone, A-DCB was significantly associated with a higher risk of primary patency [RR = 1.16 (95% CI = 1.07–1.26); *P* < 0.001; *I*^2^ = 20.9%, *P*-heterogeneity = 0.221] and lower risk of TLR [RR = 0.61 (95% CI = 0.46–0.81); *P* < 0.001; *I*^2^ = 0%, *P*-heterogeneity = 0.475]. Subgroup analysis showed that only directional, rotational, and laser atherectomy increased the probability of primary patency, but only rotational atherectomy decreased the risk of TLR. Regarding secondary outcomes, A-DCB was substantially associated with a lower likelihood of bailout stenting, any amputation, and major amputation, as well as higher ankle brachial index (ABI) following follow-up duration. Meta-regression analysis suggested that pre-intervention s (*p* = 0.015) and pre-intervention Rutherford classification (*p* = 0.038) were significantly affected the association between A-DCB and primary patency. Begg's funnel plot and Egger's test analyses indicated no publication bias in this meta-analysis.

**Conclusions:**

The addition of atherectomy improves primary patency and reduces the risk of TLR with similar safety outcomes.

**Systematic Review Registration:**

www.crd.york.ac.uk/prospero/display_record.php?ID=CRD42022382831, PROSPERO (CRD42022382831)

## Introduction

Lower extremity artery disease (LEAD) places a significant burden on healthcare, as it not only affects patients’ quality of life but also increases morbidity and mortality (1). LEAD has a global prevalence of approximately 200 million cases, which saw a 25% increase between 2000 and 2010 (2). The clinical manifestations of LEAD range from asymptomatic cases to life-threatening limb ischemia that requires immediate revascularization. According to the 2017 European Society of Cardiology guidelines, endovascular intervention is the primary treatment for revascularizing short femoropopliteal atherosclerotic lesions less than 25 cm in length. Endovascular intervention is also recommended for patients with long femoropopliteal lesions (≥25 cm) who are at high surgical risk or contraindicated for surgery (3).

Several endovascular approaches have been developed for treating LEAD, including plain balloon angioplasty, drug-coated balloon (DCB) angioplasty, drug-eluting stents (DES), and atherectomy. Recent meta-analyses have demonstrated that drug-coated balloon angioplasty (DCB) is superior to both plain balloon angioplasty and drug-eluting stents (DES) in reducing the risk of target lesion revascularization (TLR) in patients with femoropopliteal artery disease (4, 5). However, the safety and efficacy of atherectomy followed by DCB angioplasty for treating LEAD remain uncertain. Therefore, this meta-analysis aims to systematically compare the safety and efficacy of atherectomy combined with DCB angioplasty vs. DCB angioplasty alone in patients with LEAD.

## Methods

### Protocol and registration

This meta-analysis was registered in the PROSPERO (International Prospective Register of Systematic Reviews) database under the registration number CRD42022382831. It was conducted in accordance with the Preferred Reporting Items for Systematic Reviews and Meta-Analyses (PRISMA) guidelines (6).

### Search strategy

**Figure 1:**
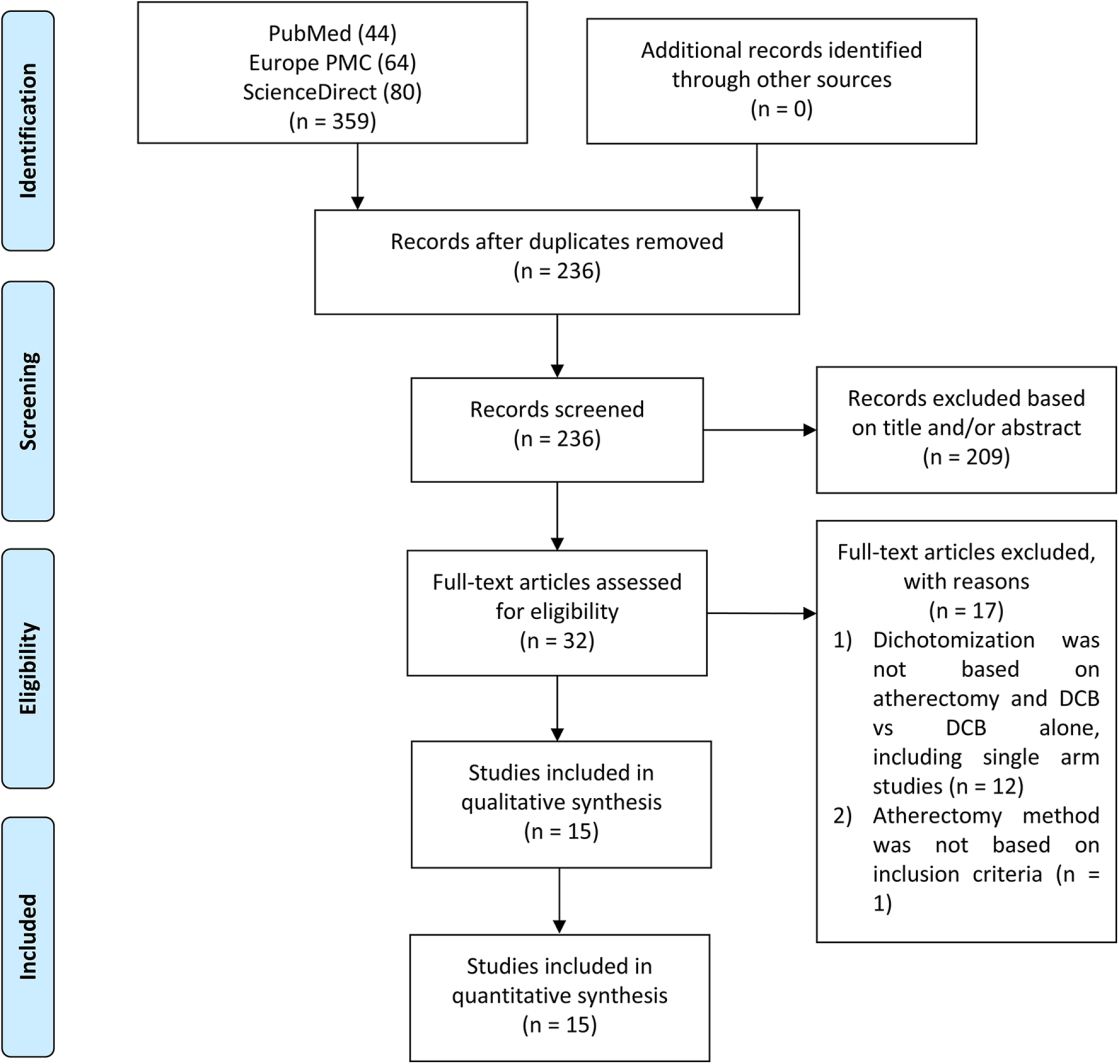
Flowchart of study selection process.

Two independent authors (RP and ICSP) conducted a systematic literature search across multiple electronic databases, including MEDLINE via PubMed, Europe PMC, and ScienceDirect, up until February 21st, 2024. The search utilized keywords such as “atherectomy” and “drug-coated balloon angioplasty” or “drug-diluted balloon angioplasty.” The search strategy did not include specific outcomes to capture a broader range of studies. The search was not limited by article type or publication date. All retrieved studies were compiled using Mendeley, and duplicate records were removed. Eligibility screening was performed manually based on titles and abstracts, followed by full-text reviews to select the studies for analysis. Discrepancies between the two authors were resolved by a third author. The search and screening process followed PRISMA guidelines, as illustrated in Figure 1.

### Eligibility criteria

Cohort and controlled trial studies comparing the efficacy and safety of atherectomy combined with DCB angioplasty vs. DCB angioplasty alone were included. The atherectomy procedures could be directional, orbital, or rotational. These studies had to report at least one outcome of interest. Studies with categorical data (events per total) or numerical data (mean and standard deviation) on the outcomes were included. Exclusion criteria were review papers, editorials, comments, case reports/series, cross-sectional studies, meta-analyses, conference abstracts, and studies not written in English.

### Data extraction, outcomes measures, and quality assessment

Two independent authors extracted the necessary data from the selected studies using a predesigned table, capturing the author's name, publication year, country, study design, total participants, age, sex, lesion type, atherectomy procedure, and follow-up duration.

Primary outcomes included primary patency and target lesion revascularization. Primary patency was defined as the absence of significant restenosis (>50% stenosis, as measured by duplex ultrasound with a peak systolic velocity ratio >2–2.5) and the absence of TLR (7–11). Target lesion revascularization was indicated for patients with LEAD symptoms related to significant restenosis or a decrease in ABI > 0.15 in the target lesion during follow-up (10, 12, 13). Secondary outcomes included all-cause mortality (ACM), amputation (major and any amputation), post-procedural complications (such as distal embolization, dissection, and perforation), technical success rate, and clinical characteristics (ABI, Rutherford category, WIQ pain score, WIQ walking distance score, WIQ walking speed score, and WIQ stair climbing score).

Quality assessment was performed using the Newcastle-Ottawa Scale (NOS) for cohort studies and the Cochrane Risk of Bias (RoB) 2.0 tool for clinical trials (14, 15). Discrepancies were resolved through discussion.

### Statistical analysis

Both categorical and numerical variables were incorporated into the meta-analysis, with event rates and means (± standard deviation) converted into risk ratios (RR) and standardized mean differences (SMD), respectively, with 95% confidence intervals (CI). A restricted-likelihood random-effects meta-analysis was conducted regardless of the heterogeneity index. A two-sided *P*-value of <0.05 was considered statistically significant. Inter-study heterogeneity was assessed using the inconsistency index (*I*^2^), with an *I*^2^ value >50% or *P*-value <0.05 indicating substantial heterogeneity. In cases of significant heterogeneity, sensitivity analysis using the leave-one-out method was employed to identify the study contributing to the heterogeneity.

Subgroup analysis was conducted to examine the impact of different atherectomy techniques (directional vs. orbital/rotational) on the primary outcomes. Meta-regression analysis was used to explore potential confounding factors. Publication bias was assessed qualitatively using Begg's funnel plot and quantitatively using Egger's test. All statistical analyses were performed using STATA version 16.0.

## Results

### Study selection and baseline characteristics

The initial literature search from three databases yielded 359 studies, of which 236 remained after the duplication removal process. After screening abstracts, titles, and full texts, 15 articles were included in this meta-analysis. Figure 1 presents a flowchart of the study selection process.

Among the 15 included studies, five were randomized controlled trials (RCTs), one was a prospective cohort study, and nine were retrospective cohort studies. Geographically, five studies were conducted in Europe, three in the United States (USA), one was a multi-country study, and the remaining studies were from Asia. In total, 1,385 participants were included, with a mean age of 68.7 ± 8.9 years, 74.2% of whom were male. The mean follow-up duration was 16 months. Regarding lesion sites, eight studies focused on femoropopliteal arteries, four on infrapopliteal arteries, one on the isolated popliteal artery, and two on femoral arteries. Concerning the atherectomy procedures, seven studies employed directional atherectomy, two used orbital atherectomy, three used laser atherectomy, and three performed rotational atherectomy. The baseline characteristics of all included studies are summarized in Table 1.

**Table 1:** Baseline characteristics of included studies.

Authors, year	Country	Study design	Total population	Age (mean ± SD)	Male (%)	CLI (%)	Lesion location	TASC A (%)	Atherectomy procedure and device	DCB device	Primary outcomes	Follow-up duration	RoB
Gandini (16)	Italy	Randomized controlled trial	48	72.1 ± 9.4	81.3	100	Superficial femoral artery	NR	LA (Turbo Elite™)	Freeway (Paclitaxel)	Primary patency, TLR	12 months	Low RoB*
Foley (7)	USA	Retrospective cohort	89	67.5 ± 8.1	93.3	30	Femoropopliteal arteries	89.2	OA (Diamondback 360°)	IN.PACT Admiral (Paclitaxel)	Primary patency, TLR	12 months	7
Stavroulakis (9)	Germany	Retrospective cohort	72	70 ± 9	52.8	36	Popliteal artery	NR	DA (TurboHawk, SilverHawk, Pantheris, and HawkOne)	IN.PACT Admiral (Paclitaxel), Freeway (Paclitaxel), Lutonix, and Passeo Lux (Paclitaxel)	Primary patency, TLR	12 months	7
Zeller (12)	Multi country	RCT	102	69 ± 8.9	66.7	1.7	Femoropopliteal arteries	39.7	DA (SilverHawk TurboHawk)	Cotavance (Paclitaxel)	Primary patency, TLR	12 months	Low RoB*
Stavroulakis (8)	Germany	Retrospective cohort	47	71 ± 9	55.3	52.8	Common femoral artery	NR	DA (TurboHawk HawkOne)	IN.PACT Admiral (Paclitaxel), Passeo Lux (Paclitaxel)	Primary patency, TLR	12 months	7
Kim (17)	South Korea	Retrospective cohort	59	67.1 ± 9.4	64.4	8	Femoropopliteal arteries	30.5	RA (Diamondback 360°, Phoenix, JetStream)	NR	Primary patency, TLR	12 months	7
Cai (10)	China	Randomized controlled trial	94	67 ± 10	76.6		Femoropopliteal arteries	85	DA (SilverHawk TurboHawk)	Orchid (Paclitaxel)	Primary patency, TLR	24 months	Low RoB*
Kokkinidis (18)	USA	Retrospective cohort (PSM)	113	69.7 ± 7.9	97.3	18	Femoropopliteal arteries	NR	OA (Diamondback 360°)	IN.PACT Admiral (Paclitaxel)	Primary patency, TLR	24 months	8
Bohme (19)	Germany	Randomized controlled trial	61	67.7 ± 9.7	70.5	13	Femoropopliteal arteries	NR	LA (Turbo Elite™, Turbo-Booster™, and Turbo Tandem™)	IN.PACT Admiral (Paclitaxel), and IN.PACT Pacific (Paclitaxel)	Primary patency, TLR	24 months	Low RoB*
Rastan (20)	Switzerland	Randomized controlled trial	80	72.1 ± 8.3	76.3	72	Infrapopliteal arteries	77.5	DA (SilverHawk TurboHawk)	Lutonix (Paclitaxel)	Primary patency, TLR	12 months	Low RoB*
Rodoplu (13)	Turkey	Retrospective cohort	121	61.2 ± 9.7	67.8	74	Femoropopliteal arteries	76	RA (Phoenix)	NR	Primary patency, TLR	24 months	7
Yang (21)	China	Retrospective cohort	79	68.4 ± 6.45	79.7	100	Infrapopliteal arteries	NR	LA (NR)	NR	Primary patency, TLR	24 months	
Kavala (22)	Turkey	Retrospective cohort	226	65.2 ± 9.7	73.9	0	Femoropopliteal arteries	82.3	DA (Phoenix)	Lovix (Paclitaxel)	Primary patency	24 months	8
Rodoplu (23)	Turkey	Retrospective cohort	128	66.4 ± 10.7	67.2	74.2	Infrapopliteal arteries	NR	RA (Phoenix)	Biopath (Paclitaxel)	Primary patency, TLR	24 months	7
Zeller (11)	USA	Randomized controlled trial	66	75 ± 7.8	77.3	73	Infrapopliteal arteries	45.7	DA (NR)	Lutonix (Paclitaxel)	Primary patency, TLR	12 months	Low RoB*

CLI, critical limb ischemia; DA, directional atherectomy; DCB, drug-coated balloon; LA, laser atherectomy; OA, orbital atherectomy; PSM, propensity score matched; RA, rotational atherectomy; SD, standard deviation; TASC, trans-atlantic inter-society consensus; TLR, target lesion revascularization.

* Evaluated using cochrane risk of bias assessment tool, the nature of the studies precludes adequate blinding.

### Meta-analysis of atherectomy combined with DCB angioplasty and primary outcomes

**Figure 2:**
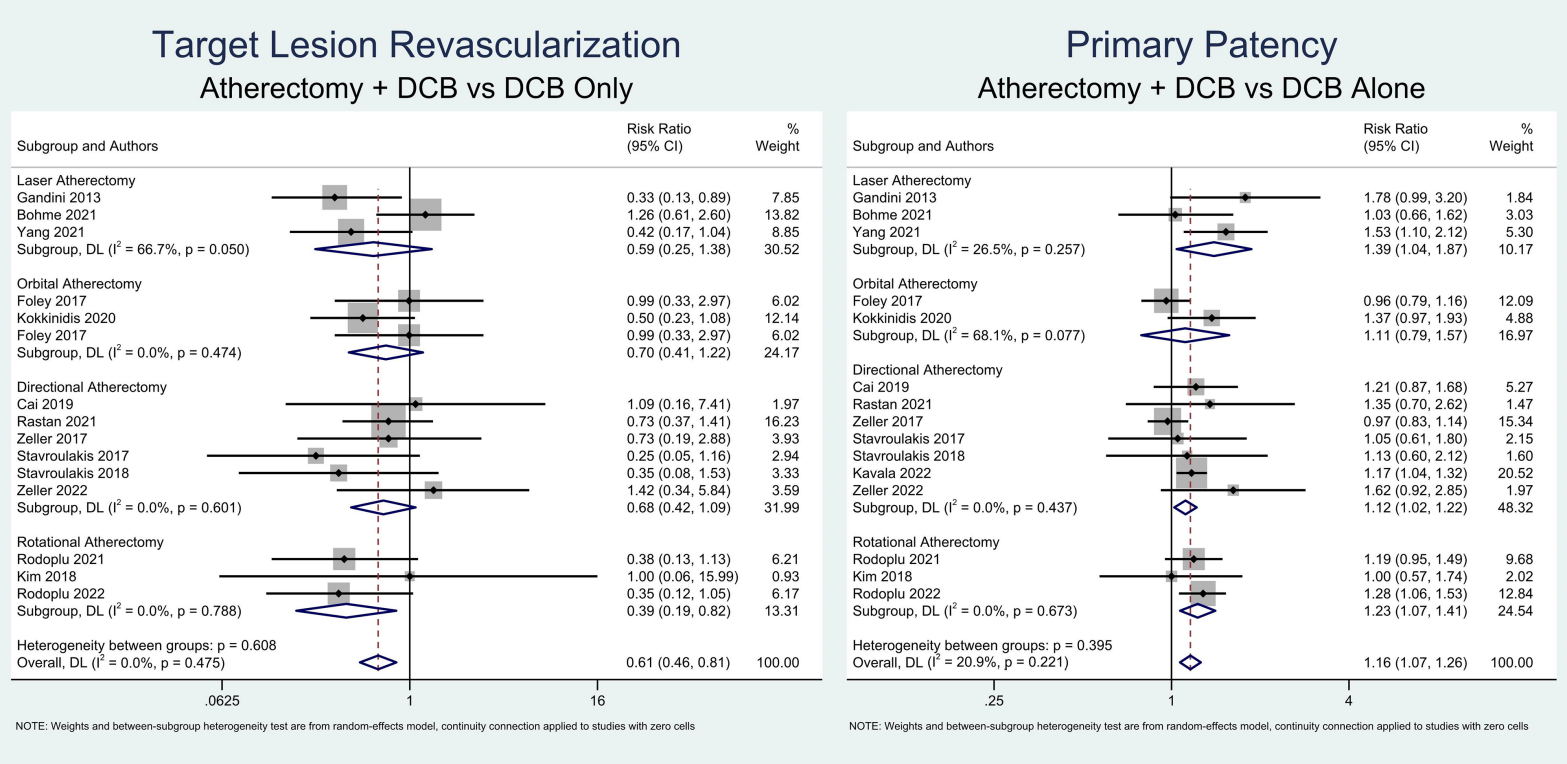
Meta-analysis of atherectomy followed by DCB angioplasty and primary outcomes.

This meta-analysis found that atherectomy followed by DCB angioplasty was significantly associated with a higher likelihood of primary patency (RR = 1.16; 95% CI = 1.07–1.26; *P* < 0.001; *I*^2^ = 20.9%, *P*-heterogeneity = 0.221) and a lower risk of TLR (RR = 0.61; 95% CI = 0.46–0.81; *P* < 0.001; *I*^2^ = 0%, *P*-heterogeneity = 0.475). Due to low heterogeneity, sensitivity analysis was not performed. A meta-analysis of the primary outcomes is depicted in Figure 2.

Subgroup analysis based on the atherectomy approach revealed that directional atherectomy (RR = 1.12; 95% CI = 1.02–1.22; *P* = 0.013; *I*^2^ = 0%, *P*-heterogeneity = 0.426), rotational atherectomy (RR = 1.23; 95% CI = 1.07–1.41; *P* = 0.004; *I*^2^ = 0%, *P*-heterogeneity = 0.675), and laser atherectomy (RR = 1.39; 95% CI = 1.04–1.86; *P* = 0.025; *I*^2^ = 25.6%, *P*-heterogeneity = 0.261) significantly increased the probability of primary patency. However, orbital atherectomy did not show a significant increase in primary patency (RR = 1.11; 95% CI = 0.79–1.57; *P* = 0.543; *I*^2^ = 68.2%, *P*-heterogeneity = 0.076). Furthermore, only rotational atherectomy significantly reduced the likelihood of TLR (RR = 0.39; 95% CI = 0.19–0.83; *P* = 0.014; *I*^2^ = 0%, *P*-heterogeneity = 0.788). In contrast, directional atherectomy (RR = 0.67; 95% CI = 0.42–1.08; *P* = 0.100; *I*^2^ = 0%, *P*-heterogeneity = 0.595), orbital atherectomy (RR = 0.63; 95% CI = 0.33–1.17; *P* = 0.144; *I*^2^ = 0%, *P*-heterogeneity = 0.318), and laser atherectomy (RR = 0.59; 95% CI = 0.25–1.39; *P* = 0.288; *I*^2^ = 66.2%, *P*-heterogeneity = 0.052) did not show significant reductions in TLR.

### Meta-analysis of atherectomy combined with DCB angioplasty and secondary outcomes

The meta-analysis of secondary outcomes is summarized in Tables 1–3. Atherectomy combined with DCB angioplasty significantly reduced the risk of bailout stenting (RR = 0.50; 95% CI = 0.36–0.69; *P* < 0.001; *I*^2^ = 0%, *P*-heterogeneity = 0.561), any amputation (RR = 0.56; 95% CI = 0.36–0.86; *P* = 0.008; *I*^2^ = 0%, *P*-heterogeneity = 0.541), major amputation (RR = 0.35; 95% CI = 0.17–0.69; *P* = 0.003; *I*^2^ = 0%, *P*-heterogeneity = 0.77), and improved ankle-brachial index (SMD = 0.24; 95% CI = 0.05–0.44; *P* = 0.013; *I*^2^ = 44.6%, *P*-heterogeneity = 0.081). However, compared to DCB angioplasty alone, atherectomy followed by DCB angioplasty was not associated with significant differences in all-cause mortality, technical success, dissection, distal embolization, perforation, Rutherford category, or WIQ walking scores for speed, distance, stair climbing, or pain.

**Table 2:** Pooled findings of meta-analysis of secondary outcomes in categorical variables.

Outcomes	Risk ratio/standardized mean difference (95% CI); *p*-value	Heterogeneity index; *p*-value	Number of studies
All-cause mortality	0.888 (0.592–1.333); *p* = 0.567	16.9%; *p* = 0.274	13
Technical success	1.039 (0.999–1.082); *p* = 0.057	45.9%; *p* = 0.035	13
Bailout stenting	0.496 (0.359–0.685); *p* < 0.001	0%; *p* = 0.561	11
Major amputation	0.346 (0.173–0.691); *p* = 0.003	0%; *p* = 0.770	8
Any amputation	0.557 (0.36–0.861); *p* = 0.008	0%; *p* = 0.541	6
Dissection	0.565 (0.317–1.088); *p* = 0.053	39.9%; *p* = 0.101	9
Embolization	0.854 (0.398–1.834); *p* = 0.686	0%; *p* = 0.617	10
Perforation	1.100 (0.402–3.007); *p* = 0.853	0%; *p* = 0.688	8
Rutherford category > 1	0.761 (0.530–1.092); *p* = 0.138	13.2%; *p* = 0.316	3

**Table 3:** Pooled findings of meta-analysis of secondary outcomes in numerical variables.

Outcomes	SMD (95% CI); *p*-value	Heterogeneity index; *p*-value	Number of studies
ABI	0.243 (0.051–0.435); *p* = 0.013	44.6%; *p* = 0.081	8
WIQ walking distance score (%)	−0.067 (−0.347–0.214); *p* = 0.641	46.4%; *p* = 0.133	4
Rutherford category	0.196 (−0.038–0.429); *p* = 0.1	0%; *p* = 0.977	3
WIQ walking speed score (%)	0.008 (−0.196–0.211); *p* = 0.940	0%; *p* = 0.737	3
WIQ stair climbing (%)	0.122 (−0.556–0.8); *p* = 0.724	75.3%; *p* = 0.044	2
WIQ pain score (%)	0.00 (−0.22–0.22); *p* = 1.00	0%; *p* = 1.00	2

### Meta-Regression analysis

Meta-regression analysis showed that factors such as age, sex, smoking status, hypertension, diabetes mellitus, heart failure, myocardial infarction, coronary artery disease, hyperlipidemia, chronic kidney disease, cerebrovascular disease, chronic obstructive pulmonary disease, pre-intervention ABI, lesion length, vessel diameter, stenosis diameter, Trans-Atlantic Inter-Society Consensus (TASC) classification, vessel runoff, lesion characteristics (in-stent restenosis, chronic total occlusion, and *de novo* lesion), and severe calcification did not significantly affect the association between atherectomy combined with DCB angioplasty and TLR (*p* > 0.05). However, pre-intervention ABI (*p* = 0.015) and pre-intervention Rutherford classification (*p* = 0.038) were significant factors affecting the association between atherectomy followed by DCB angioplasty and primary patency.

### Publication bias and risk of bias assessment

**Figure 3:**
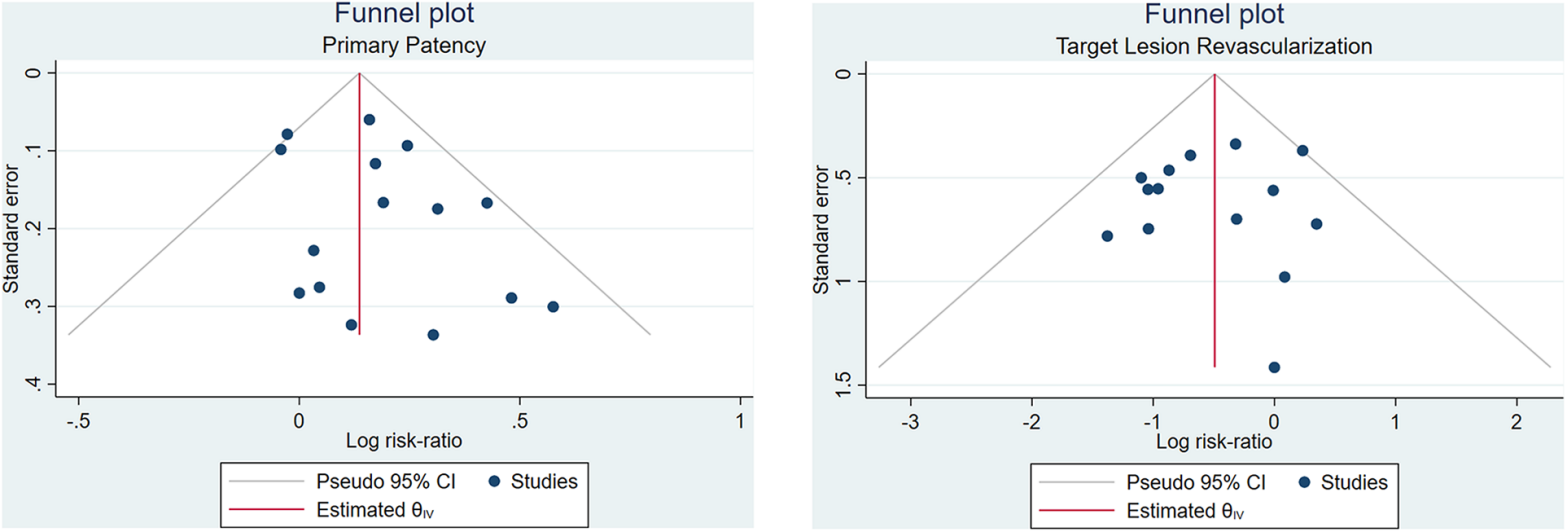
Funnel plot analysis for primary patency and TLR.

Begg's funnel plot analysis was performed for six outcomes, revealing that primary patency and technical success rate had asymmetrical funnel plots, while TLR (Figure 3), all-cause mortality, bailout stenting, and distal embolization showed symmetrical funnel plots. Additionally, Egger's test showed no small study effects for any of the outcomes (*p* > 0.05), indicating no publication bias in this meta-analysis.

## Discussion

**Figure 4:**
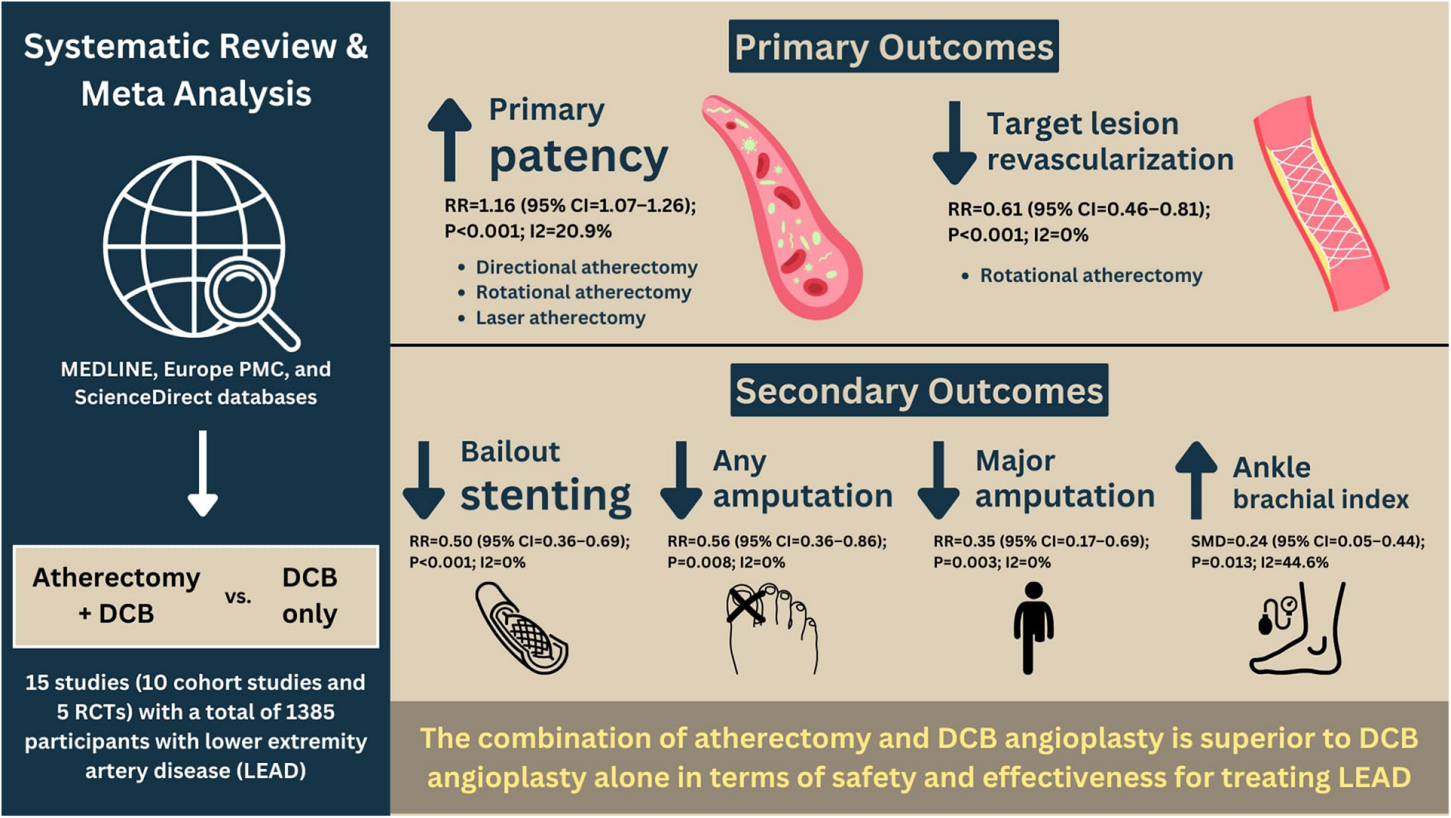
Central illustration.

In this meta-analysis, atherectomy followed by DCB angioplasty was associated with a higher likelihood of achieving primary patency and a reduced risk of TLR (Figure 4). Further subgroup analysis indicated that atherectomy subtypes outperformed DCB alone in terms of primary outcomes; however, orbital atherectomy did not achieve primary patency. There was no significant difference between the groups in all-cause mortality, technical success, dissection, distal embolization, perforation, Rutherford category, WIQ walking speed, WIQ walking distance, WIQ stair climbing, or WIQ pain score at the end of follow-up. Meta-regression analysis also revealed that confounding factors did not affect the results.

The mechanisms through which DCB and atherectomy work to resolve obstruction in LEAD will be further discussed. DCB is a relatively new approach in percutaneous interventional treatment. Paclitaxel, the active drug in the device, is commonly used to prevent hyperplasia of the blood vessel walls. Its lipophilic and hydrophobic properties allow for quick absorption by endothelial cells, enabling the rapid delivery of an effective drug dose. Paclitaxel remains in the vessel walls for an extended period, preventing restenosis (24–26). However, the effectiveness of DCB in severely calcified lesions remains uncertain.

Treating heavily calcified arteries in the lower limbs can be challenging with endovascular techniques. Calcified lesions may recoil after angioplasty, preventing adequate artery expansion and leading to residual stenosis and treatment failure. Severe calcification in LEAD patients is an independent predictor of late lumen loss, which may negate the anti-proliferative effects of DCB. Therefore, removing non-compliant plaque components before DCB angioplasty is essential to optimize drug delivery by reducing the calcium burden (27).

Theoretically, atherectomy devices can reduce intimal calcifications and high plaque loads, enhancing drug delivery and improving DCB efficacy. Atherectomy decreases the risk of perforation and dissection by applying minimal pressure to the artery wall and distributing the drug more evenly. This is supported by the lower rates of acute outcomes (such as bailout stenting) and improved primary patency observed in the A-DCB group compared to DCB alone (7, 28). Our meta-analysis aligns with these findings, showing higher ABI recovery rates and fewer amputations at follow-up in the A-DCB group.

In terms of safety, atherectomy combined with DCB demonstrated similar rates of adverse events and complications compared to DCB alone. Some complications warrant further investigation but appear to be preventable. First, the widespread adoption of distal protection devices in atherectomy with DCB is highly recommended, as these devices are effective in preventing embolization during the procedure. This is evidenced by the comparable embolization rates across both groups. Second, vessel wall damage caused by the atherectomy device may contribute to the occurrence of perforations (10), though the overall complication rates between the two groups did not differ significantly.

One notable point is the lack of primary patency observed with orbital atherectomy. The mechanism of action of atherectomy devices is complex, making it difficult to directly compare outcomes or recommend specific devices for certain lesion characteristics. This discrepancy may be due to the small number of studies included in the orbital atherectomy category. Additionally, the degree of severe calcification differed significantly between groups in studies using orbital atherectomy (84% vs. 40.5%), likely due to selection bias. Severe calcification is an independent predictor of restenosis, which may explain the similar primary patency rates between the groups (7, 29).

Several limitations in this meta-analysis should be noted. First, most studies had a follow-up duration of up to 12 months, with a maximum of 24 months, so long-term outcomes remain unclear. Second, there were not enough randomized controlled trials (RCTs) to provide strong evidence. Third, the use of various atherectomy and DCB devices may have influenced the pooled outcomes in unexpected ways. Finally, the clinical relevance of these findings may be limited by the variability in atherectomy techniques and patient populations across the included studies.

## Conclusions

This meta-analysis suggests that atherectomy followed by DCB angioplasty improves primary patency and reduces the risk of TLR. However, it does not significantly affect all-cause mortality, Rutherford category, WIQ walking speed, WIQ walking distance, WIQ stair climbing, or WIQ pain score at the end of follow-up. Furthermore, the addition of atherectomy does not reduce the incidence of technical success, dissection, distal embolization, or perforation. Meta-regression analysis indicated that the confounding factors analyzed did not significantly impact the pooled effect estimate.

## Data Availability

The original contributions presented in the study are included in the article/Supplementary Material, further inquiries can be directed to the corresponding author.
